# Homonymous Quadrantanopsia as the First Manifestation of Cerebral Metastasis of Invasive Mole: a case report

**DOI:** 10.1186/1752-1947-6-117

**Published:** 2012-04-24

**Authors:** De-Lu Song, Yong Zhong, Feng Feng, Yuan Li, Meng-Hui Li

**Affiliations:** 1 Department of Ophthalmology, Peking Union Medical College Hospital, Chinese Academy of Medical Sciences & Peking Union Medical College, 1# Shuaifuyuan, Beijing 100730, China; 2 Scheie Eye Institute, University of Pennsylvania Perelman School of Medicine. Philadelphia, PA, USA; 3 Department of Radiology, Peking Union Medical College Hospital, Chinese Academy of Medical Sciences & Peking Union Medical College, Beijing, China; 4 Department of Pathology, Peking Union Medical College Hospital, Chinese Academy of Medical Sciences & Peking Union Medical College, Beijing, China; 5 Department of Gynecology and Obstetrics, Peking Union Medical College Hospital, Chinese Academy of Medical Sciences & Peking Union Medical College, Beijing, China

**Keywords:** Cerebral metastasis, Homonymous Quadrantanopsia, Invasive mole

## Abstract

**Introduction:**

Homonymous quadrantanopsia results from retrochiasmal lesions in the visual pathway. Invasive mole is a benign tumor that arises from myometrial invasion of a hydatidiform mole via direct extension through tissue or venous channels. Cerebral metastasis of invasive mole is rare and there has been no report demonstrating homonymous quadrantanopsia as the first manifestation of metastasis in any trophoblastic neoplasms.

**Case presentation:**

We report the case of a 31-year-old Asian woman who presented with right homonymous inferior quadrantanopsia from the mass effect of a solitary cerebral metastasis from an invasive mole. A magnetic resonance image (MRI) of the brain showed a metastatic tumor in the left occipital lobe. The visual field improved slightly after chemotherapy. There was a reduction in the tumor size and the surrounding edema. This is the first case report demonstrating that homonymous quadrantanopsia should be included in the manifestations of the metastasis of an invasive mole.

**Conclusions:**

The presentation of homonymous quadrantanopsia must alert ophthalmologists to conduct a complete medical history and arrange specialist consultation.

## Introduction

Homonymous hemianopia is usually secondary to stroke, head trauma, or tumors [1]. Homonymous quadrantanopsia is due to retrochiasmal lesions in the visual pathway. Invasive mole is a benign tumor that arises from myometrial invasion of a hydatidiform mole via direct extension through tissue or venous channels. Approximately 10% to 17% of hydatidiform moles will result in invasive mole [2]. Cerebral metastasis of invasive mole is rare and there has been no report demonstrating homonymous quadrantanopsia as the first manifestation of metastasis in any trophoblastic neoplasms. We report the case of a patient who presented with right homonymous inferior quadrantanopsia from the mass effect of a solitary cerebral metastasis of an invasive mole.

## Case presentation

**Figure 1:**
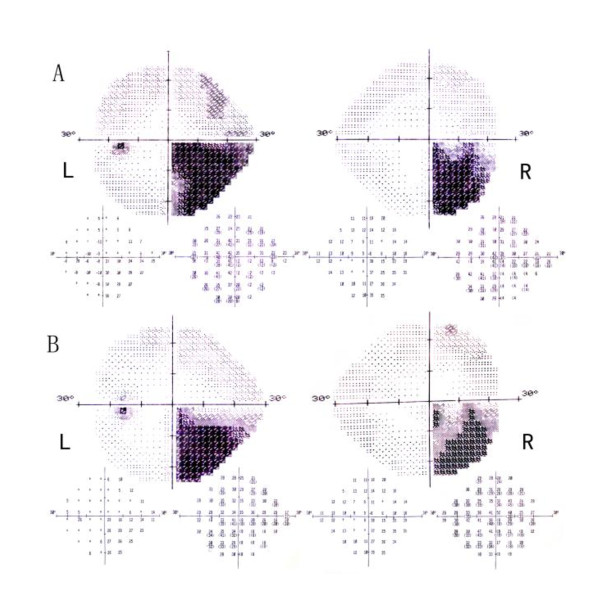
**A (L: left eye; R: right eye) Humphrey visual field demonstrating a congruous right homonymous inferior quadrantanopsia**. B Repeat visual field test showed slight improvement.

**Figure 2:**
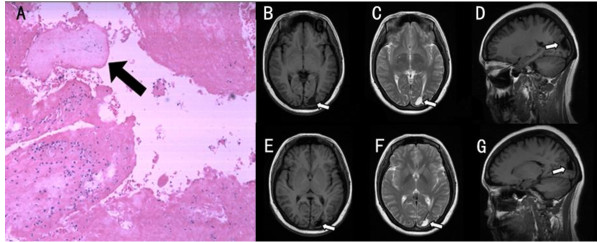
**A, Pathological examination showed extensive coagulation necrosis of myometrium and degenerative chorionic villus (arrow, hematoxylin-eosin stain, original magnification ×60)**. B. D, Axial and sagittal T1-weighted brain MRI showing the low-intensity metastatic lesion (arrow). C, T2-weighted image demonstrating the high-signal mass. E. G, After chemotherapy, the low-intensity metastatic lesion diminishes on axial and sagittal T1-weighted image and edema subsided compared with pre-chemotherapy. F, The high-intensity mass also shrinks on T2-weighted image.

A 31-year-old Asian woman who complained of 'loss of right side vision', headache, dizziness, nausea, paroxysmal eye pain, and blurred vision was seen in our clinic. Humphrey visual field testing with a 4-mm^2 ^Goldmann size III stimulus (0.43°diameter) on a dim background (31.5apostilb) revealed a right homonymous inferior quadrantanopsia (Figure 1A). Her medical history was significant with dilatation and curettage for menolipsis and irregular vaginal bleeding two years ago. Pathological examination of her curettage specimen showed hydatidiform mole. Microscopic examination demonstrated that molar vesicles had penetrated deeply into the myometrium giving rise to extensive coagulation necrosis and residual degenerative chorionic villus (Figure 2A). On eye examination, the best corrected visual acuity was 20/20 OU. Pupillary responses were normal with no relative afferent pupillary defect. Extraocular eye movements were normal and she did not have any history of ocular diseases. A funduscopic examination showed no papilledema or retinal hemorrhage. Other cranial nerve functions were within normal limits. The remainder of the ophthalmologic examination was all negative.

On medical examination, an ultrasonic test of her pelvis showed 1.0 × 2.0 cm^2 ^echogenic dots in the anterior wall of the lower uterine segment. An MRI of her brain revealed a low signal lesion at the left occipital lobe on a T1-weighted image (Figure 2B, D) and high signal mass on a T2-weighted image (Figure 2C). An MRI of her chest and abdomen demonstrated no evidence of systemic metastasis. Laboratory tests showed that her serum human chorionic gonadotropin (hCG) level was elevated to 78,187.5 mIU/mL. General gynecologic examination was negative. After hospitalization, this patient was treated with ten cycles of chemotherapy including intrathecal chemotherapy with methotrexate (MTX) and intravenous injection of vincristine, 5-fluorouracil (5-FU), cyclophosphamide and etoposide. After the first cycle of chemotherapy, her symptoms related to the increased intracranial pressure disappeared. Her vision field improved slightly after the second cycle of chemotherapy (Figure 1B), but showed no further improvement until the completion of ten cycles of the chemotherapy program. A repeat MRI of the brain demonstrated a reduction in the left occipital lobe metastasis and the associated edema. The patient was left with a permanent lesion (Figure 2E, F, G).

## Discussion

Hydatidiform mole refers to an abnormal pregnancy characterized by varying degrees of trophoblastic proliferation and vesicular swelling of placental villi associated with an absent or an abnormal fetus and or embryo [3]. It has been reported that the hydatidiform moles that erode the wall of the uterus, burrow into the myometrium and may even burst though the uterus into the peritoneum are called invasive moles [4,5]. Local metastasis and invasion is common for invasive moles. Metastasis may occur in the lung, pelvis and vagina. Rare sites include the gastrointestinal tract, spleen, and kidney. Central nervous system metastasis is rare. It is often fatal because of the high risk of intracerebral hemorrhage, neurological deterioration, and death [4]. Invasive mole is often diagnosed clinically rather than pathologically based on persistent hCG elevation after molar evacuation and is frequently treated with chemotherapy without a histopathologic diagnosis [2]. Therefore, the intracerebral lesion in our patient should be attributed to the metastasis of an invasive mole. The usual clinical interpretation of homonymous quadrantanopsia is the effect of lesions of the optic radiations course between the optic tract and the striate cortex. Superior homonymous defects are generally associated with temporal lobe lesions, whereas inferior defects commonly result from lesions of the parietal lobe. Cerebrovascular disease is a potential cause of homonymous quadrantanopsias. Rampini P *et al. *[6] presented a case of left quadrantanopsia secondary to traumatic subclavian steal syndrome. Infarction of the ventromedial aspect of the inferior occipital lobe [7] and striate cortex [8] leading to homonymous superior quadrantanopsia have been reported in the literature as well. Association with non-occlusive vascular events related to vertebrobasilar hypoperfusion rather than embolization is also not uncommon. In 1962, Smith [9] reviewed a series of homonymous hemianopia cases. He found that occipital lobe lesions are the most common cause of hemianopia and are more frequent in men than women. Vascular lesions are the most common cause of occipital lobe field defects compared to tumors. Cerebral metastasis of tumor is also a common cause leading to this kind of special visual field defect. Groom *et al. *[10] presented an optic tract syndrome case with homonymous hemianopia caused by metastasis to the lateral geniculate body and optic tract secondary to metastatic breast cancer. The pituitary gland is an uncommon site for metastasis. Baehring *et al. *[11] reported a case of heteronymous inferior quadrantanopsia of hypothalamic mass lesion with extension into the pituitary fossa.

## Conclusions

This is the first reported case of cerebral metastasis by invasive mole presenting with a homonymous inferior quadrantanopia. The presentation of homonymous quadrantanopsia must alert ophthalmologists to search for a complete medical history and specialist consultation. The key value of urgent neuroimaging in all cases of acute onset homonymous hemianopsia should be emphasized. Additionally, physicians looking after patients with hydatidiform moles should consider metastasis in patients complaining of visual symptoms. The timely diagnosis and treatment could result in a favorable outcome.

## Consent

Written informed consent was obtained from the patient for publication of this case report and any accompanying images. A copy of the written consent is available for review by the editor of this journal.

## Competing interests

The authors declare that they have no competing interests.

## Authors' contributions

DLS was the major contributor in studying the case and writing the manuscript and was involved in the medical care of the patient. YZ was the physician who admitted the patient and performed the visual field test. FF is the head of the department of radiology and responsible for the MRI reading. YL was involved in the pathological diagnosis and MHL was involved in the chemotherapy of the patient. All authors read and approved the final manuscript.
